# A new time-varying coefficient regression approach for analyzing infectious disease data

**DOI:** 10.1038/s41598-023-41551-1

**Published:** 2023-09-06

**Authors:** Juxin Liu, Brandon Bellows, X. Joan Hu, Jianhong Wu, Zhou Zhou, Chris Soteros, Lin Wang

**Affiliations:** 1 Department of Mathematics and Statistics, University of Saskatchewan, Saskatoon, S7N 5E6 Canada; 2 Department of Statistics and Actuarial Science, Simon Fraser University, Vancouver, V5A 1S6 Canada; 3 Department of Mathematics and Statistics, York University, Toronto, M3J 1P3 Canada; 4 Department of Statistical Sciences, University of Toronto, Toronto, M5G 1X6 Canada; 5 Department of Mathematics and Statistics, University of New Brunswick, Fredericton, E3B 5A3 Canada

**Keywords:** Infectious diseases, Viral infection

## Abstract

Since the beginning of the global pandemic of Coronavirus (SARS-COV-2), there has been many studies devoted to predicting the COVID-19 related deaths/hospitalizations. The aim of our work is to (1) explore the lagged dependence between the time series of case counts and the time series of death counts; and (2) utilize such a relationship for prediction. The proposed approach can also be applied to other infectious diseases or wherever dynamics in lagged dependence are of primary interest. Different from the previous studies, we focus on time-varying coefficient models to account for the evolution of the coronavirus. Using two different types of time-varying coefficient models, local polynomial regression models and piecewise linear regression models, we analyze the province-level data in Canada as well as country-level data using cumulative counts. We use out-of-sample prediction to evaluate the model performance. Based on our data analyses, both time-varying coefficient modeling strategies work well. Local polynomial regression models generally work better than piecewise linear regression models, especially when the pattern of the relationship between the two time series of counts gets more complicated (e.g., more segments are needed to portray the pattern). Our proposed methods can be easily and quickly implemented via existing R packages.

## Introduction

Since the WHO declared the novel coronavirus (COVID-19) outbreak a global pandemic on March 11, 2020, impacts of the pandemic on people’s daily life have been profound in many different aspects (e.g., physical health, mental health, social impacts). Despite the strong global desire to end the pandemic, the evolving variants and subvariants of SARS-CoV-2 have posed challenges for predicting what is ahead. Under the pressure of co-circulation of viral infections, such as the tripledemic (COVID-19, seasonal influenza, RSV) in the 2022–2023 influenza season, healthcare systems can be easily over-burdened due to the quickly rising number of cases with severe symptoms in need of medical care. Though the currently dominant Omicron subvariants can be less severe than the original variant, in early 2023 the Canadian healthcare system remained under the risk of crisis. This was due to the risk that the faster transmission/spread of the dominant variants may lead to a larger number of people who need to seek medical care within a short time period.

There is a large body of existing literature on predicting/forecasting COVID-19-related deaths and hospitalizations. According to Avery et al.^1^, two primary types of modeling are dominant. One type of model is *mechanistic* and focuses on the underlying process of the disease spread. For example, system dynamics models with different formulations of state variables have seen numerous applications in COVID-19 modeling. The Centers for Disease Control and prevention (CDC) has featured a set of different prediction models for COVID-19 death forecasting^2^. These models are generally complex and rely on assumptions that are often violated (e.g. homogeneity) or hard to verify, as discussed by Avery et al.^1^ and Li et al.^3^.

The other primary type of model is *phenomenological*, usually parameterized through curve-fitting based on reported data. This is the type of modeling our proposed work follows. The focus is *not* the transmission dynamics, but rather the relationship between the reported cases and deaths. The majority of the existing work on phenomenological modeling considers a *single* time series of interest (e.g., time series of case counts). To name a few, Cascon and Shadwick^4^ and Harvey and Kattuman^5^ use the Gompertz Function to model cumulative pandemic case counts, and Dash et al.^6^ use a logistic growth model with accommodations for nonlinear trend and seasonality. Additionally, Petropoulos et al.^7^ applied a non-seasonal multiplicative error to a multiplicative trend exponential smoothing model. Change-point models have received a substantial amount of attention to capture abrupt changes in a single time series (e.g. Jiang et al.^8^ and^9^). Clearly which time series model to use should depend on the pattern exhibited by the data to be analyzed.

It is worth noting that artificial intelligence (AI) methods have seen some successful applications in COVID-19 related predictions^10,11^. Nonetheless, it is still not clear when AI methods can be applied successfully^12^ or which AI methods are best. In contrast, the aim of our proposed modeling is to find a more general and flexible tool that can accommodate various kinds of relationships between one time series and another.

There has been quite sparse literature that makes use of the relationship between the different time series for prediction. To our best knowledge, Hierro et al.^13^ is the only paper of this kind. In their work, the so-called delayed elasticity method (hereafter referred to as DEM) is used to characterize the relationship between cumulative death counts and cumulative case counts. Intuitively, their method, which is essentially classical linear regression models, may work well at the beginning of the pandemic (limited available data) but may not be able to fully capture the evolving relationships for a longer study period. We compare our proposed methods to theirs in “Data analysis” section and the supplementary document.

Distinct from the existing literature on COVID-19, we aim to build a general and flexible modeling approach that can capture the dynamic nature of the relationships between different COVID-19 data series of interest. With such a relationship, we can predict future deaths/hospitalizations based on the case counts up to present. It is worth noting that our approach can also be used to analyze other infectious disease data or wherever dynamics in a lagged dependence relationship is of interest. With this aim in mind, time-varying coefficient regression models^14^ are a natural choice. Different from the classical linear regression models, the regression coefficients are not fixed as constant but rather functions of some other covariate(s) (e.g., time). The fact that Canada, for example, has experienced several different waves driven by different coronavirus variants suggests that it would be more appropriate to consider the time-varying relationship between case counts and death counts (or hospitalization counts).

To summarize, the novelty of our work is two-fold. First, we introduce an explicit way to account for the lagged dependence between the predictor and response variables in the context of varying-coefficient models. Moreover, statistical learning is successfully combined with a machine learner that selects the optimal lag based on out-of-sample predictability. Second, our method produces inferences for the out-of-sample predictions, while most of the existing literature on time-varying coefficient models focuses on regression coefficients.

As explained in Section 2 of the seminal paper by Hastie and Tibshirani^14^, time-varying coefficient regression models have a broad general form and thus include several commonly used models as special cases (e.g., generalized linear models, generalized additive models, piecewise linear regression models). In this study, we consider two different techniques: local polynomial regression and piecewise linear regression. Influenced by the extensive literature on the kernel estimation in time-varying coefficient models, we embarked with local polynomial regressions^15,16^. Then we realized the smoothness assumption of the regression coefficients may not always be valid, especially when there are abrupt changes. For example, when Omicron’s subvariants quickly took over the dominance of Omicron in some major cities (or travel hubs), the relationship between case counts and deaths/hospitalizations may have changed quickly accordingly. To address this possibility, we also consider piecewise linear regression models that (1) bear simple parametric forms and (2) can capture abrupt changes.

We acknowledge the fact that neither the reported case counts nor the reported death counts are truly reflecting the underlying true variables, respectively. As such, our objective is to capture the dynamic relationship between *reported* COVID-19 data. One key novelty in our approach is to identify the lag between different reported data series (e.g., death counts and case counts). For details, please see “Models and notation” section.

## Models and notation

**Figure 1:**
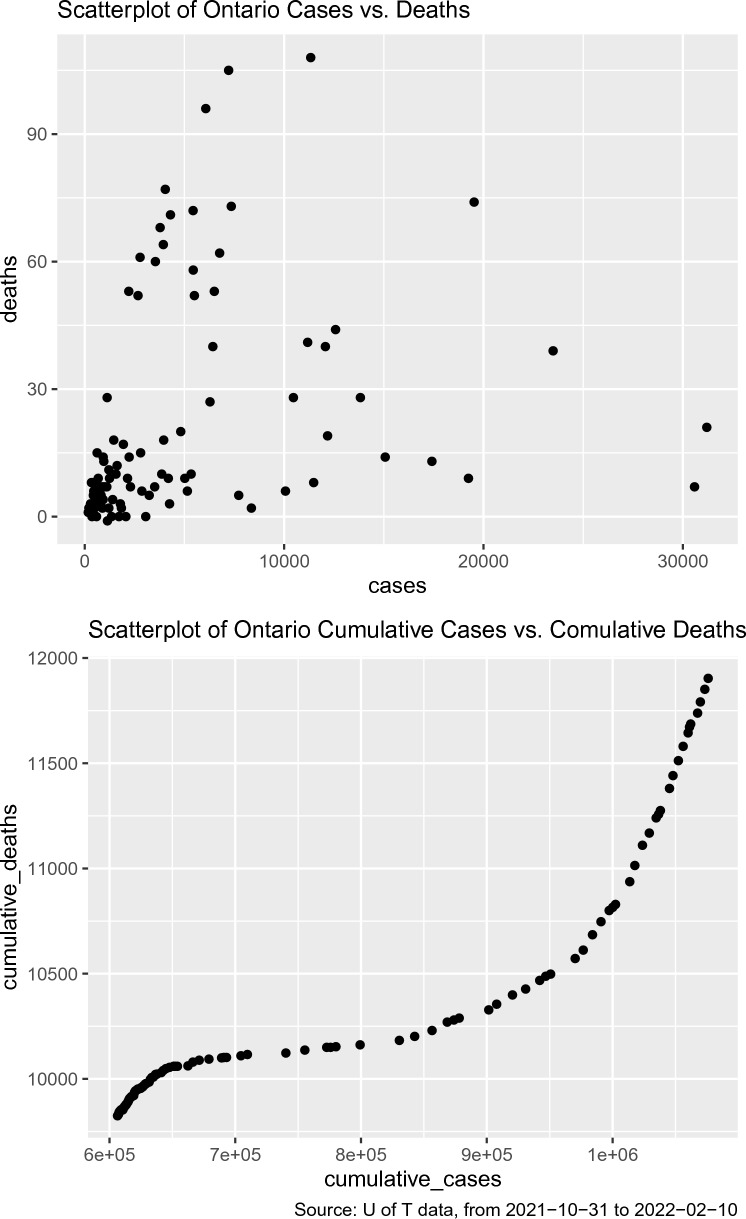
Scatter plots for Ontario case counts vs death counts (daily counts in the top panel vs cumulative counts in the bottom panel) between Oct 31, 2021 and February 10, 2022.

Modeling the daily counts is a natural choice and has been considered in many studies. Nonetheless, we noticed the pattern between cumulative counts is much cleaner. Consider, for example, Fig.  1 for the scatter plots for Ontario data between 2021-10-31 and 2022-02-10. As shown in the bottom panel in Fig.  1, there is a very neat pattern between cumulative counts. But there is no such clean pattern in the daily counts plot (top panel in Fig.  1). The noise level seems to be fairly high so even a time-varying coefficient model may not lead to a good fit. It turns out the signal may get strengthened by balancing out the noise in the daily counts when adding them up. Therefore, we consider *cumulative* counts in our modeling but utilize daily counts for model assessment/comparison. After all, the future trend of daily counts is of our primary interest. We remark that the fitted/predicted values in cumulative counts can be easily converted to fitted/predicted values in daily new counts by taking the difference between any two consecutive cumulative counts.

In the following, we will present two modeling strategies to capture the lagged dynamic relationship, that is, local polynomial regression and piecewise linear regression.

### Local polynomial regression with lagged dependence

Different from the classical regression models, the time varying coefficient regression model allows the regression coefficients $\beta$’s to change over some other covariate (called smoothing variable). Suppose the data are in form of $(Y_i, X_i)_{i=1}^n$. The time-varying coefficient regression model is

1 $$\begin{aligned} Y_{i+L}= & {} \beta _{i,0} + \beta _{i,1} X_{i} + \epsilon _i, \quad i=1,\ldots , n - L, \end{aligned}$$

where $\beta _{i,0}=\beta _0(\dfrac{i}{n})$ and $\beta _{i, 1}=\beta _1(\dfrac{i}{n})$ for some smooth function ${\varvec{\beta }}=(\beta _0,\beta _1)^\prime : [0, 1]\rightarrow {\mathbb {R}}^2$. We assume $E(\epsilon _i|X_i)=0$.

To reflect the lagged dependence between the death count time series and other time series (e.g., case count time series), the outcome variable (*Y*) in the above model is for time $i+L$ conditional on the value of the predictor (*X*) at time *i*. The optimal choice of *L* is selected based on the criterion of minimizing mean squared prediction errors for out-of-sample daily counts predictions. More details will be given in “Selection of lag” section.

The local constant (also called the Nadaraya-Watson method) and local linear estimation methods are the commonly used local polynomial methods for time-varying coefficient regression models. The local constant estimates are obtained by minimizing the following objective function, that is,

$$\begin{aligned} {\hat{{\varvec{\beta }}}}(t) = \text{ argmin}_{{\varvec{\theta }}}\sum _{i=1}^n\left( y_i-\textbf{x}^\prime _i{\varvec{\theta }}\right) ^2K_b(i/n-t), t\in [0,1], \end{aligned}$$

where $K_b(\cdot )={\displaystyle {1\over b}}K(\cdot /b)$ is the kernel and *b* is a bandwidth, and $\textbf{x}_i=(1, x_{i})^\prime$. Obviously the resulting estimator depends on the choice of bandwidth *b*. As stated in^17^, the bandwidth is selected by cross validation (leave-one-out cross-validation by default in tvReg). The triweight kernel is the default choice in tvLM, an R function in the R package tvReg^17^.

The local constant estimator can be written in the following matrix form that resembles the weighted least squares estimator. Let

$$\begin{aligned} \textbf{y} & =(y_1,\ldots ,y_n)^\prime ,\\ \textbf{X} & = (\textbf{x}_1, \textbf{x}_2,\ldots , \textbf{x}_n)^\prime ,\\ W_t & = \text{ diag }\left( K_b(1/n-t), K_b(2/n-t), \ldots , K_b(i/n-t), \ldots , K_b(1-t)\right) . \end{aligned}$$

Then we have the local constant estimator, denoted by ${\hat{{\varvec{\beta }}}}$,

2 $$\begin{aligned} {\hat{{\varvec{\beta }}}}(t)= & {} (\textbf{X}^\prime W_t \textbf{X})^{-1}\textbf{X}^\prime W_t \textbf{y}. \end{aligned}$$

Similarly, local linear estimators can be obtained by minimizing

$$\begin{aligned} ({\hat{{\varvec{\beta }}}}(t),{\hat{{\varvec{\beta }}}}^{(1)}(t)) = \text{ argmin}_{{\varvec{\theta }}_0,{\varvec{\theta }}_1}\sum _{i=1}^n\left( y_i-\textbf{x}^\prime _i{\varvec{\theta }}_0 - \textbf{x}^\prime _i{\varvec{\theta }}_1(i/n-t)\right) ^2K_b(i/n-t), \end{aligned}$$

where ${\varvec{\beta }}^{(1)}(t)$ is the first order derivative of ${\varvec{\beta }}(t)$.

Let $U_t=\hbox {diag}\{1/n-t,\ldots , i/n-t,\ldots , 1-t\}$ and $\Gamma _t=(\textbf{X}, U_t\textbf{X})$. Therefore, the local linear estimator can be expressed in the following matrix form

3 $$\begin{aligned} ({{\hat{{\varvec{\beta }}}}^\prime (t)}, {{\hat{{\varvec{\beta }}}}^{(1)\prime }(t)})^\prime= & {} (\Gamma _t^\prime W_t \Gamma _t)^{-1}\textbf{X}^\prime W_t \textbf{y}. \end{aligned}$$

### Piecewise linear regression model with lagged dependence

As explained in “Introduction” section, the smoothness imposed for the time-varying coefficients may not be able to capture some abrupt changes. Therefore, we consider piecewise linear regression models as an alternative.

4 $$\begin{aligned} Y_{i+L}= & {} \beta _0 + \beta _1x_i + \beta _2 (x_i - k_1)_{+} + \ldots + \beta _{p+1}(x_i-k_p)_{+} + \epsilon _i,\quad i=1,\dots , n - L, \end{aligned}$$

where

$$\begin{aligned} (x_i-k)_{+}=\left\{ \begin{array}{c c} x_i-k, &{}\;\text{ if } x_i-k\ge 0\\ 0,&{}\;\text{ if } x_i-k<0. \end{array} \right. \end{aligned}$$

with *k* defined as a breakpoint. We also assume $E(\epsilon _i|X_i=x_i)=0$.

In our data analysis, the R package “segmented”^18^ is used to implement the model estimation for piecewise linear regression models. The restarting bootstrap method^19^ is implemented in “segmented” to handle spurious local minima (e.g., flat segments). The segmented package also provides an automatic option^20^ for determining the number and location of the breakpoints. We found the automatic option over-estimated the number of breakpoints, which led to worse performance for out-of-sample prediction. Therefore, we don’t recommend the use of the automatic option because out-of-sample prediction is of our primary interest. In the following data analysis with the piecewise linear regression method, we selected the starting values of breakpoints by examining the scatter plot.

## Selection of lag

In this subsection, we discuss how to determine the lag for the dependence between the response variable and the covariate. For the response variable, we consider $Y_{t+l}$ with $l= L_{min}, L_{min} + 1, \ldots , L_{max}$ with varying lag *l*. In our data analysis, we use $L_{min} = 5$ and $L_{max}=21.$ The optimal lag that best captures the lagged dependence, denoted by *L*, is selected to be the one that gives smallest mean squared prediction error for out-of-sample daily counts prediction.

To explain, for each $l\in \{L_{min}, \ldots , L_{max}\}$, we fit the model (either local polynomial regression or piecewise linear regression) for the same training data. Suppose the maximum date of the data is $T_{m}$, then the training data set consists of data up to date $T_m - 2L_{max}$. Then we predict the cumulative death counts for the time window $[n-2L_{max} + 1, n- 2L_{max} + l]$. We refer to the next section for details about how to conduct out-of-sample prediction. By subtracting any two consecutive cumulative counts, we get the predicted daily counts for the time window $[n - 2L_{max} + 2, n - 2L_{max} + l]$. Therefore, we can calculate the mean squared prediction error (MSPE) for *daily* death counts reported during $[n -2L_{max} + 2, n - 2L_{max} + l]$ for each *l*. The optimal lag is the value of *l* that yields the smallest MSPE.

## Prediction

One immediate potential application of the lagged dependence structure discussed in “Models and notation” section is for prediction. In piecewise linear regression models, the prediction is fairly straightforward conditional on the estimated breakpoints. Basically we make use of the simple linear regression model for the segment that the new observations of the predictor fall into.

In the local polynomial regression setting, we consider the Direct-recursive hybrid multi-step forecast^21^ and can be implemented in the function *forecast()* in the R package tvReg. Here is the outline of how the prediction can be done.

Step 1. Apply the local polynomial regression method to the data until time point *n*.
Step 2. Predict $Y_{n+1}$ by using the estimate of the regression coefficient ${\varvec{\beta }}_n$ from Step 1, that is, ${\hat{Y}}_{n+1}={{\hat{{\varvec{\beta }}}}}^\prime _n\textbf{x}_{n+1}$.
Step 3. Predict $Y_{n+2}$ by treating ${\hat{Y}}_{n+1}$ as if it were the actual observation at time $n + 1$ and implementing the local polynomial regression method to the augmented data $(\textbf{x}_i, y_i), i=1,\ldots , n+1$ where $y_{n+1}={\hat{Y}}_{n+1}$. Then we repeat Steps 2 and 3 until *L* future predictions is done, where *L* is the lag discussed in the previous section.

**Figure 2:**
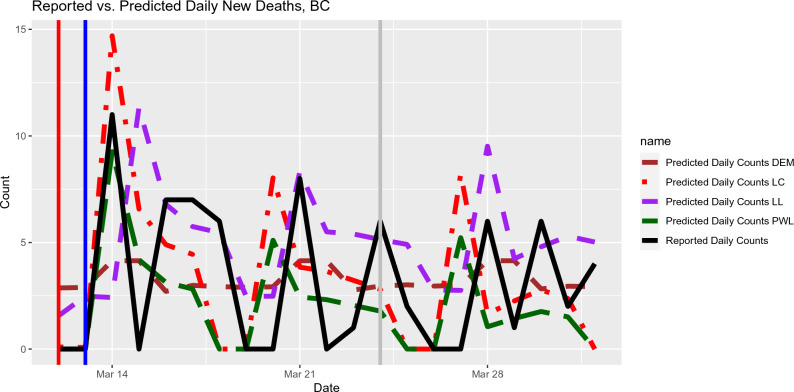
Out-of-sample Prediction for daily deaths in BC based on the input data from December 5, 2021 to April 1, 2022. The Mean Squared Prediction Errors (MSPE) for the out-of-sample predictions and selected lags (in brackets) are listed as follows. Local Constant: 17 (20 days), Local Linear: 16 (21 days), Piecewise Linear: 13 (20 days), DEM: 7 (9 days).

The potential problem with this prediction strategy is that it uses the predicted values as if they were the real observations. If the regression coefficients change very slowly over a short prediction time window, such a prediction strategy may not be a big problem. As shown in all the figures except Fig. 2, local constant regression models seem to be the winner for the out-of-sample daily counts prediction. It is worth noting that the propagated error in using the predicted values may lead to unreliable results. As shown in Fig. 5, the predicted daily deaths based on local linear method tends to deviate more from the reported counts near the end of the out-of-sample prediction window.

In the following, we present bootstrap methods for calculating point-wise and simultaneous confidence bands for out-of-sample predictions in time-varying coefficient regression models. The assumption is i.i.d. random error terms in time-varying coefficient regression models.

### Point-wise confidence bands for out-of-sample predictions

The $100(1-\alpha )\% (0<\alpha <1)$ point-wise confidence bands of $Y(u), u\in [{\displaystyle {n+1\over n+L}}, 1]$ are defined by

$$\begin{aligned} {[}{\hat{Y}}(u)-c_{\alpha /2}\times sd({\hat{Y}}(u)|{\mathbb {D}}), {\hat{Y}}(u)+c_{\alpha /2}\times sd({\hat{Y}}(u)|{\mathbb {D}})], \end{aligned}$$

where ${\mathbb {D}}=(z_1, z_2, \ldots , z_n, X_1, X_2, \ldots , X_n)$. Please note that $z_i=i/n, Y(z_i)=Y_i,$ and $X(z_i)=X_i$. Using a similar rational as that in^22^ for constructing confidence intervals for regression coefficients, we implement the following steps to construct the confidence intervals for the predicted values.

Step 1. For available data (say up to time *n*), we fit a time-varying regression model and produce the fitted values ${\hat{y}}_i$ and residuals $e_i = y_i-{\hat{y}}_i, i=1,\ldots , n.$
Step 2. Generate synthetic data $y^*_i = {\hat{y}}_i +e^*_i,$ where $e^*_i = \eta _i{\tilde{e}}_i,$ and ${\tilde{e}}_i=e_i{\displaystyle {1\over n}}\sum _{i=1}^ne_i$, that is, the centred residuals. Re-fit the time-varying regression model based on the synthetic data and use the built-in R function *forecast()* to predict the future *L* observations, denoted by ${\hat{y}}^*_{n+1}, \ldots , {\hat{y}}^*_{n+L}$, or equivalently, $\hat{y^*}({\displaystyle {n+1\over n+L}}), \hat{y^*}({\displaystyle {n+2\over n+L}}) \ldots , \hat{y^*}(1)$.
Step 3. Repeat Step 2 for *B* times and obtain *B* bootstrap predicted values for the future *L* observations. For $u\in [{\displaystyle {n+1\over n+L}}, 1]$, the estimate of $sd({\hat{Y}}(u)|{\mathbb {D}})$ is the sample standard deviation of bootstrap samples $\{{\hat{y}}^{*(b)}(u): b=1, \ldots , B\}$ and is denoted by $sd^*({\hat{Y}}(u))$.
Step 4. For each $b=1,\ldots , B$, we calculate
  $$\begin{aligned} Q^{*(b)}(u)= & {} {\displaystyle {\hat{y^*}_{u, b}-{\hat{y}}_{u}\over sd^*(\hat{y^*}_{u})}}. \end{aligned}$$
  The estimate of $c_{\alpha /2}(u), u\in [{\displaystyle {n+1\over n+L}}, 1]$ is the upper $\alpha /2$ percentile of $\{Q^{*(b)}(u)\}$.

### Simultaneous confidence bands for out-of-sample predictions

Since the point-wise confidence bands only provide interval estimates for each given future time point, we here discuss simultaneous confidence bands that allow us to infer all future time points simultaneously. As such, one can make inference about the trend of future predictions based on such confidence bands. Following the bootstrap methods proposed by^23^, we construct the simultaneous confidence bands as outlined below.

Let

$$\begin{aligned} Q=\sup _{u\in \left[ {\displaystyle {n+1\over n+L}}, 1\right] } {\displaystyle {|\hat{y(u)}-y(u)|\over sd(\hat{y(u)}|{\mathbb {D}})}}, \end{aligned}$$

where $${\mathbb {D}}=(u_1,\ldots , u_n, X(u_1), \ldots , X(u_n))$$ with $$u_i={\displaystyle {i\over n}}$$.

The $100(1-\alpha )\%$ simultaneous confidence band for $\{E(Y(u)|{\mathbb {D}})\}$ for ${u\in [{\displaystyle {n+1\over n+L}}, 1]}$ is in the form of

$$\begin{aligned} \hat{y(u)}\pm sd(Y(u)|{\mathbb {D}})C_{\alpha /2}. \end{aligned}$$

The last two terms after the ± can be estimated from the bootstrap methods.

Step 1. Fit a time-varying coefficient regression model. Denote the predicted values by $\hat{y(u)}, u\in [{\displaystyle {n+1\over n+L}}, 1].$ The predicted values can be directly calculated by using the built-in R function *forecast()* in the R package *tvReg*.
Step 2. For each $i=1,\ldots , n$, generate a bootstrap sample
  $$\begin{aligned} y^*_i={\hat{y}}_i +{\tilde{e}}_i\eta _i, \end{aligned}$$
  where ${\tilde{e}}_i=e_i-{\displaystyle {1\over n}}\sum _{i=1}^ne_i$, that is, centred residuals; $\eta _i{\mathop {\sim }\limits ^{i.i.d.}} N(0, 1)$.
Step 3. Repeat Step 2 *m* times to get a size m sample for $\left( {\hat{y}}({\displaystyle {n+1\over n+L}}), {\hat{y}}({\displaystyle {n+2\over n+L}}), \ldots , {\hat{y}}(1)\right)$, denoted by
  $$\begin{aligned} \left( {\hat{y}}^{*(k)}({\displaystyle {n+1\over n+L}}), {\hat{y}}^{*(k)}({\displaystyle {n+2\over n+L}}), \ldots , {\hat{y}}^{*(k)}(1)\right) , \; k=1,2,\ldots , m. \end{aligned}$$
  Based on the *m* samples, we estimate $sd(Y(u)|{\mathbb {D}})$ by the corresponding sample standard deviations, denoted by $sd^*(Y(u)|{\mathbb {D}})$.
Step 4. Repeat Step 2 *M* times to get a size *M* sample for $\left( {\hat{y}}({\displaystyle {n+1\over n+L}}), {\hat{y}}({\displaystyle {n+2\over n+L}}), \ldots , {\hat{y}}(1)\right)$. Calculate
  $$\begin{aligned} Q^*_s=\sup _{u\in [{\displaystyle {n+1\over n+L}}, 1]} {\displaystyle {|{\hat{y}}^{*(s)}(u)-{\hat{y}}(u)|\over sd^*({\hat{Y}}(u)|{\mathbb {D}})}}, s=1,2,\ldots , M. \end{aligned}$$
  Please be noted that $Q^*_s$ are bootstrap sample of *Q*.
Step 5. Use the upper $\alpha /2$ sample percentile of $\{Q^*_s: s=1,2,\ldots , M\}$ to estimate $C_{\alpha /2}$, the upper $\alpha /2$ percentile of *Q*.

## Data analysis

### Provincial data in Canada

**Figure 3:**
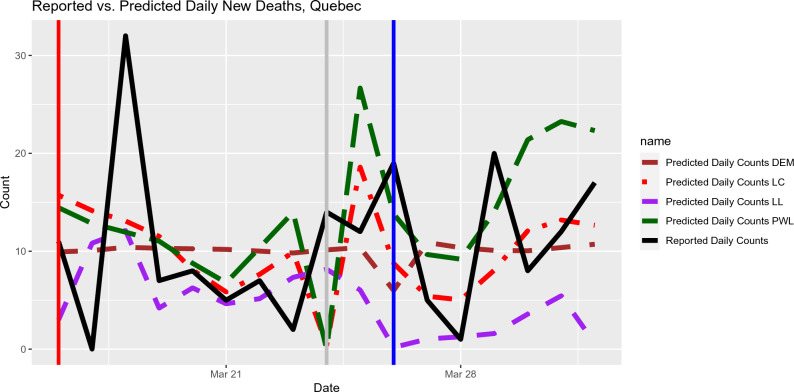
Out-of-sample Prediction for daily deaths in Quebec based on the input data from Oct 31, 2021 to April 1, 2022. The Mean Squared Prediction Errors (MSPE) for the out-of-sample predictions and selected lags (in brackets) are listed as follows. Local Constant: 43 (7 days), Local Linear: 102 (17 days), Piecewise Linear: 69 (7 days), DEM: 56 (9 days).

**Figure 4:**
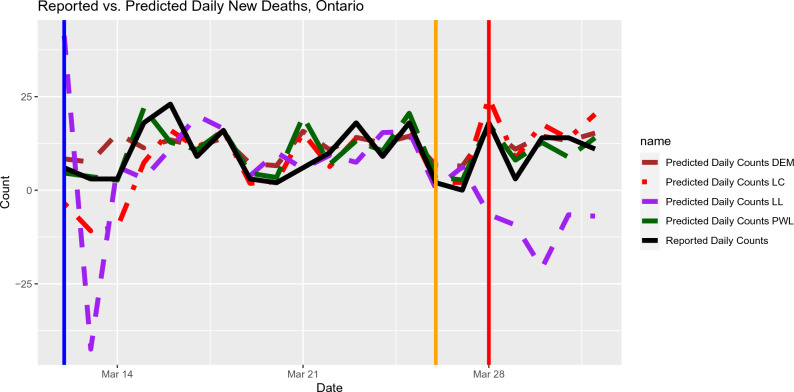
Out-of-sample Prediction for daily deaths in Ontario based on the input data from Oct 31, 2021 to April 1, 2022. The Mean Squared Prediction Errors (MSPE) for the out-of-sample predictions and selected lags (in brackets) are listed as follows. Local Constant: 25 (7 days), Local Linear: 541 (5 days), Piecewise Linear: 20 (21 days), DEM: 16 (7 days).

We use the publicly available data resource maintained by the COVID-19 Canada Open Data Working Group^24^. We consider the time window near to the first Omicron wave for provinces in Canada. Please note that the time window is different for different provinces. For each of Figs.  2, 3, 4, we plotted out-of-sample predictions for daily new deaths using local polynomial and piecewise linear regression models. The actual reported deaths were overlayed in each plot so the prediction accuracy can be easily visualized. The general impression is that local constant regression and piecewise linear regression produced better daily predictions. Local linear regression tends to be more sensitive to aberrant data points (such as negative values of some daily new deaths due to retrospective re-assessment), as suggested by Fig. 4. Moreover, local linear regression did not work for some provinces (e.g., Saskatchewan) due to some singular fits. It is likely related to the sparsity in the data due to rareness of deaths in such provinces. It is also worth noting that the starting values for the breakpoints affects the predictive performance of the piecewise linear models. In Fig. 3, the piecewise linear regression performed the worst. Because we did not set up the last breakpoint properly; the segmented package does not allow the value of breakpoints go beyond 95% percentile of the predictor values (when the sample size is larger than 20). For such circumstances, we recommend local polynomial methods.

### World-wide data: reported death counts

We use the publicly available data^25^ for analyzing country-level data. COVID-19-related deaths and hospitalizations in select countries were investigated using data spanning from the start of the Omicron wave in each country until December 31, 2022 or the last date for daily death/case counts being reported (whichever comes earlier). Cumulative counts of reported cases, deaths and hospitalizations are used in model development to reduce differences between countries with different reporting periods and frequencies. The starting value of breakpoints for the piecewise-linear models (needed for using the segmented function) were manually determined by visual inspection of the scatter plot of cumulative deaths versus cumulative cases.

**Figure 5:**
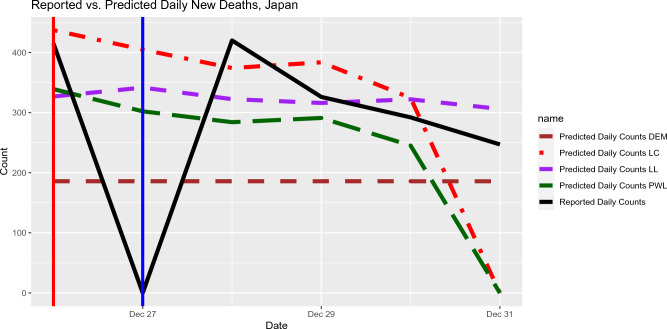
Out-of-sample Prediction for daily deaths in Japan based on the input data from January 10, 2022 to December 31, 2022. Mean Squared Prediction Errors (MSPE) for the out-of-sample predictions and selected lags (in brackets) are listed as follows. Local Constant: 46,277 (5 days), Local Linear: 23,073 (6 days), Piecewise Linear: 34,834 (5 days), DEM: 29,644 (6 days).

**Figure 6:**
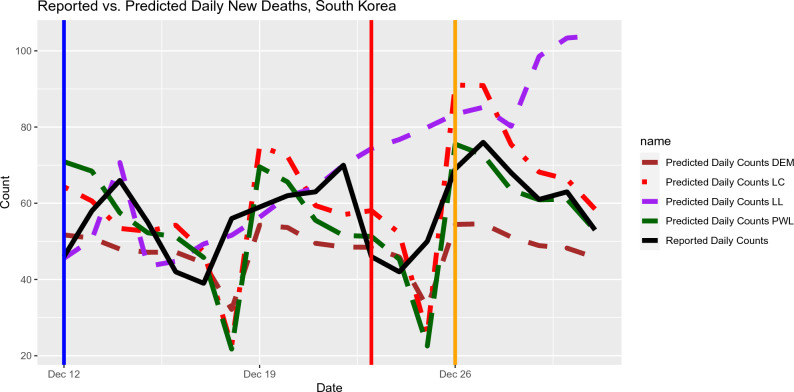
Out-of-sample Prediction for daily deaths in South Korea based on the input data from January 30, 2022 to December 31, 2022. The Mean Squared Prediction Errors (MSPE) for the out-of-sample predictions and selected lags (in brackets) are listed as follows. Local Constant: 142 (6 days), Local Linear: 997 (9 days), Piecewise Linear: 175 (20 days), DEM: 258 (6 days).

Both local polynomial regression and piecewise-linear regression produce strong predictive accuracy when used to predict COVID-19-related deaths in Japan and South Korea (Figs.  5 and  6). The success of predictions in these countries is likely due to the high quality of the data: Japan and South Korea report death counts daily and small day-to-day variation is reported.

Unlike Japan and South Korea, most other countries do not report death counts daily. The irregular spacing between observations degrades the performances of both types of models. For more data analyses on other countries, please refer to the supplementary document.

**Figure 7:**
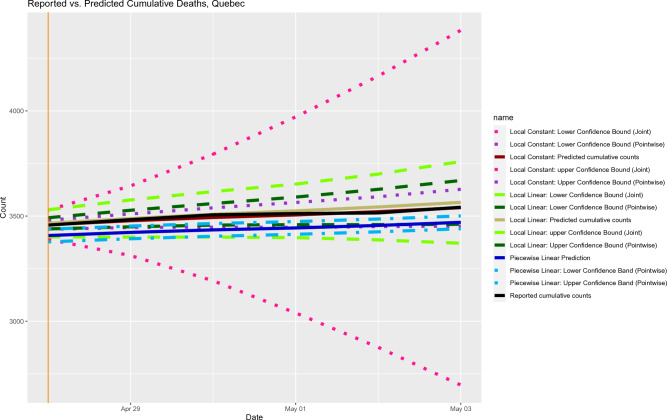
Out-of-sample prediction for cumulative deaths with pointwise and joint confidence bands.

**Figure 8:**
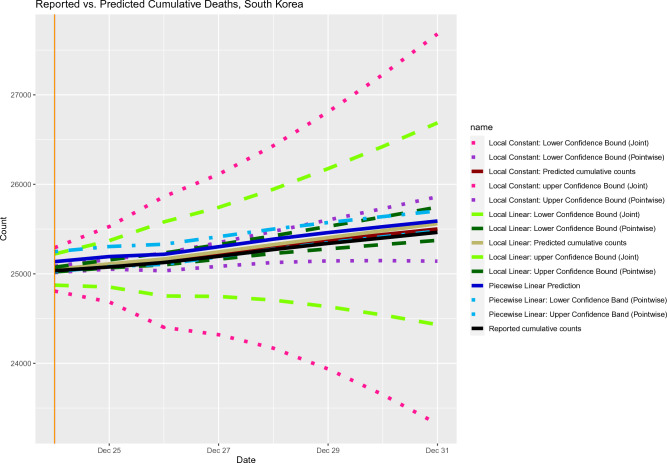
Out-of-sample prediction for cumulative deaths with pointwise and joint confidence bands.

Using the bootstrapping methods mentioned in “Point-wise confidence bands for out-of-sample predictions” and “Simultaneous confidence bands for out-of-sample predictions”, we calculated the pointwise and simultaneous confidence bands for cumulative death counts for Quebec and South Korea, respectively. As shown in Figs. 7 and 8, the simultaneous confidence bands based on local constant and local linear methods can be very wide (like trumpet near the end of the prediction window). For such a case, the trend of future data cannot be inferred from the simultaneous confidence bands.

## Summary and discussion

In this paper, we have proposed two different types of time-varying coefficient models to characterize the dynamic nature of the lagged dependent relationship between the time series of cumulative death count and the time series of cumulative case count. The value of the lag in the dependence is selected based on minimizing the mean squared prediction error for *daily* death counts. Both local polynomial regression and piecewise linear regression work well when the relationship exhibits simple patterns (such as one change point in the piecewise linear regression setting). Caution should be exercised when using the local linear method for data sets containing outliers (e.g., the negative reported daily death counts in Ontario data). The predictive performance of local linear method seems to be more sensitive to outliers, as shown in Fig. 4. Thus the proposed methods can provide a potential prediction approach as a complementary tool to the existing literature on predicting deaths/hospitalizations. The R scripts for implementing the proposed methods are posted on GitHub (https://github.com/JuxinLiu/COVID-19-data-analysis).

When the pattern gets more complicated (e.g., when the study period is longer or some rapid changes happen), local polynomial regression works better in terms of smaller out-of-sample prediction errors. We make just a quick note here that the estimation for piecewise linear regression models was implemented by using the R package *segmented*. The performance of the model estimation relies on the choice of the starting values for the breakpoints to be estimated.

We also compared the proposed methods with the Delayed Elasticity Model^13^. Based on the MSPE, our methods outperform DEM for most regions (more data analysis results are given in the supplementary document). For regions where DEM performs slightly better (Ontario and BC), the selected lag, i.e., prediction window is pronouncedly shorter.

In summary, we have developed a general and flexible modeling approach for death predictions. Model fit can be easily implemented by an R package tvReg. Based on our data analyses, the proposed method works well for most regions. Nonetheless, there are some limitations of our approach, which lead to some potential directions for future work. First, our proposed methods were designed for regular time series (e.g., daily or weekly) without missing values. But often real-world data contain missing values. For example, the Saskatchewan government changed the frequency of reporting from daily to weekly to monthly. Second, our proposed methods rely on the assumption of independent and identically distributed random error terms. If the examination of residuals show evidence of violation of such an assumption, more realistic models are needed to account for dependent random errors. Third, the confidence bands (either pointwise or joint/simultaneous) in our work refer to predicted *cumulative* counts. Ideally we will need to convert the current prediction bands (pointwise or joint) to the prediction intervals for future daily counts. An alternative way could be building the models for daily counts and then naturally the prediction is for daily counts as well^9^.

### Supplementary Information

Supplementary Information.

## Data Availability

The datasets analysed in this manuscript are publicly available in the following repositories: https://github.com/ccodwg/Covid19Canada and https://ourworldindata.org/coronavirus.
