# Supplementary material for: A new time-varying coefficient regression approach for analyzing infectious disease data
Source: Sci Rep. 2023 Sep 6;13:14687. doi: 10.1038/s41598-023-41551-1 (PMC10482960; doi:10.1038/s41598-023-41551-1)
Supplement: Supplementary file 1 — Supplementary Information. [file 41598_2023_41551_MOESM1_ESM.pdf]

A new time-varying coefficient regression approach for analyzing infectious disease data
Additional Data Analysis

Juxin Liu[1, *], Brandon Bellows[1], X. Joan Hu[2], Jianhong Wu[3], Zhou Zhou[4], Chris Soteros[1], and Lin Wang[5]

[1]Department of Mathematics and Statistics, University of Saskatchewan, Saskatoon, S7N 5E6, Canada
[2]Department of Statistics and Actuarial Science, Simon Fraser University, Vancouver, V5A 1S6 Canada
[3]Department of Mathematics and Statistics, York University, Toronto, M3J 1P3, Canada
[4]Department of Statistical Sciences, University of Toronto, Toronto, M5G 1X6, Canada
[5]Department of Mathematics and Statistics, University of New Brunswick, Fredericton, E3B 5A3, Canada
[*]liu@math.usask.ca

ABSTRACT

Additional data analysis and accompanying plots are presented to supplement the methodologies and conclusions described in the main paper.

1 Canadian Province Results

The local constant method works (slightly) better than the local linear method for Manitoba, New Brunswick, and Nova Scotia data. Meanwhile, the piecewise linear regression method performs best for New Brunswick, Newfoundland and Labrador, and Nova Scotia data.

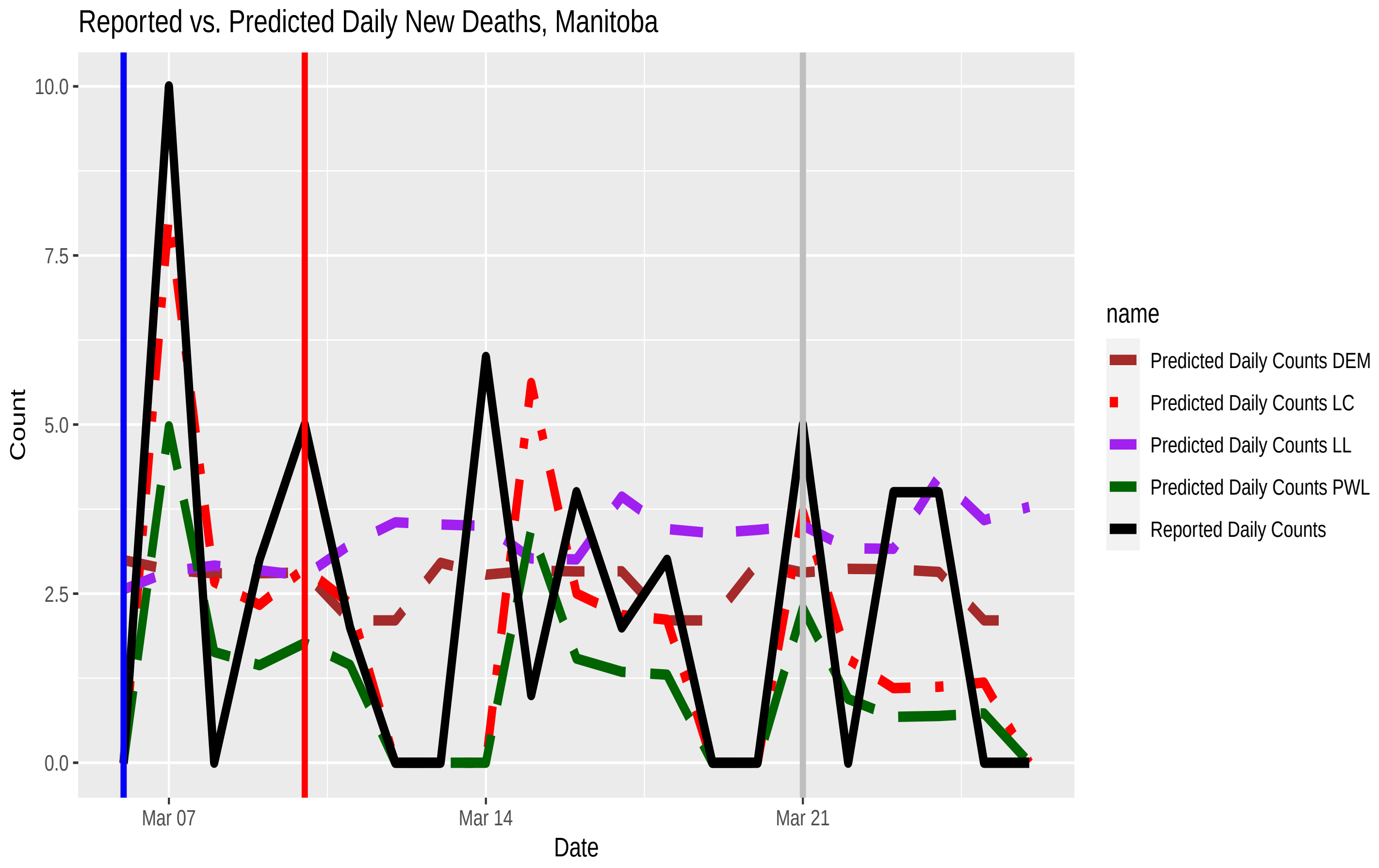

Figure S.1. Prediction for Manitoba daily reported death counts based on the input data from Dec 5, 2021 to Mar 26, 2022. Vertical lines indicate the start of predictions for each type of model. Mean Squared Prediction Errors (MSPE) for the out-of-sample predictions are as follows. Local Constant: 5 (21 days), Local Linear: 6 (17 days), Piecewise Linear: 6 (21 days), DEM: 4 (6 days).

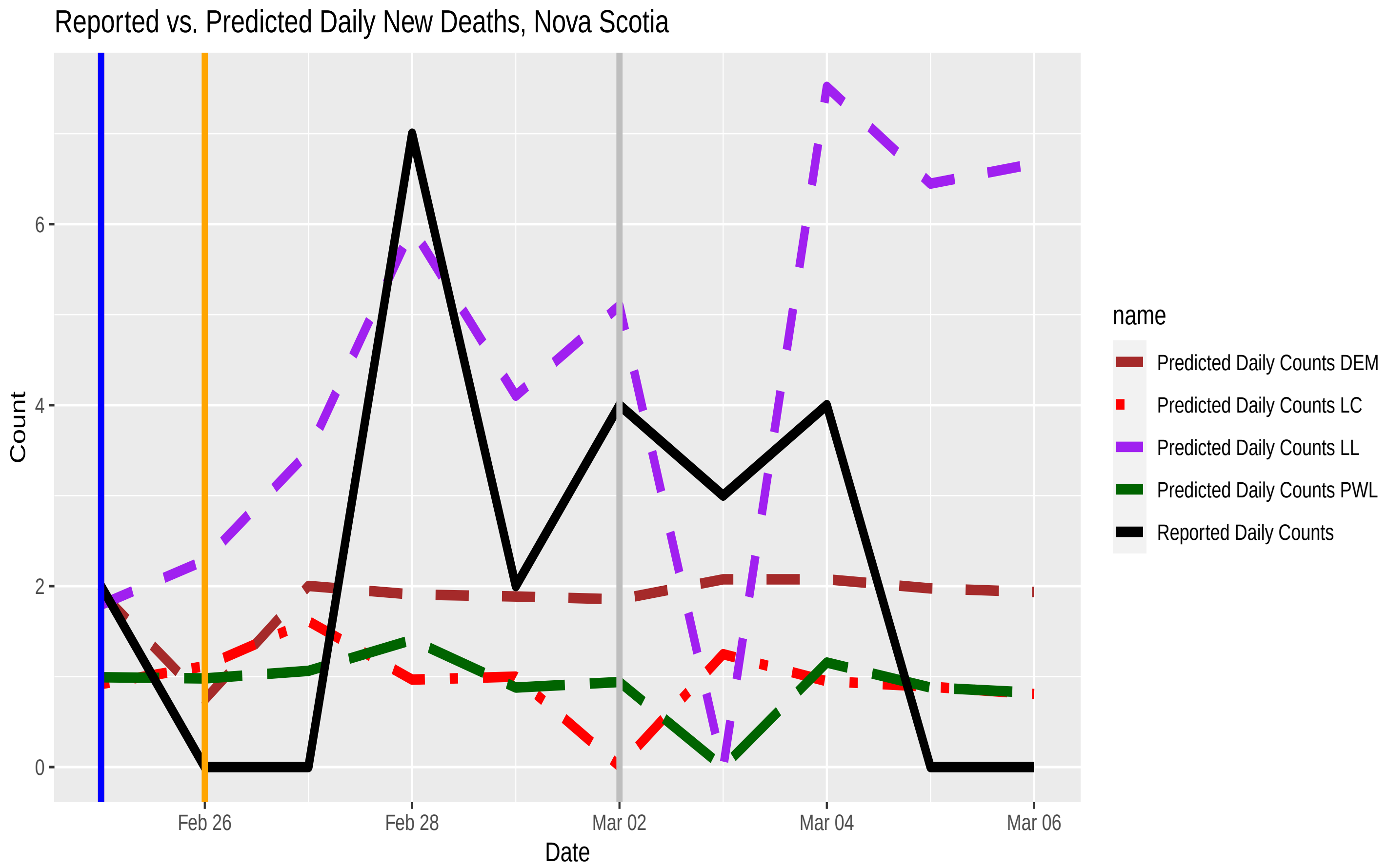

Figure S.2. Prediction for Nova Scotia daily reported death counts based on the input data from Dec 5, 2021 to Mar 6, 2022. Vertical lines indicate the start of predictions for each type of model. Mean Squared Prediction Errors (MSPE) for the out-of-sample predictions are as follows. Local Constant: 8 (9 days), Local Linear: 13 (10 days), Piecewise Linear: 6 (10 days), DEM: 5 (5 days).

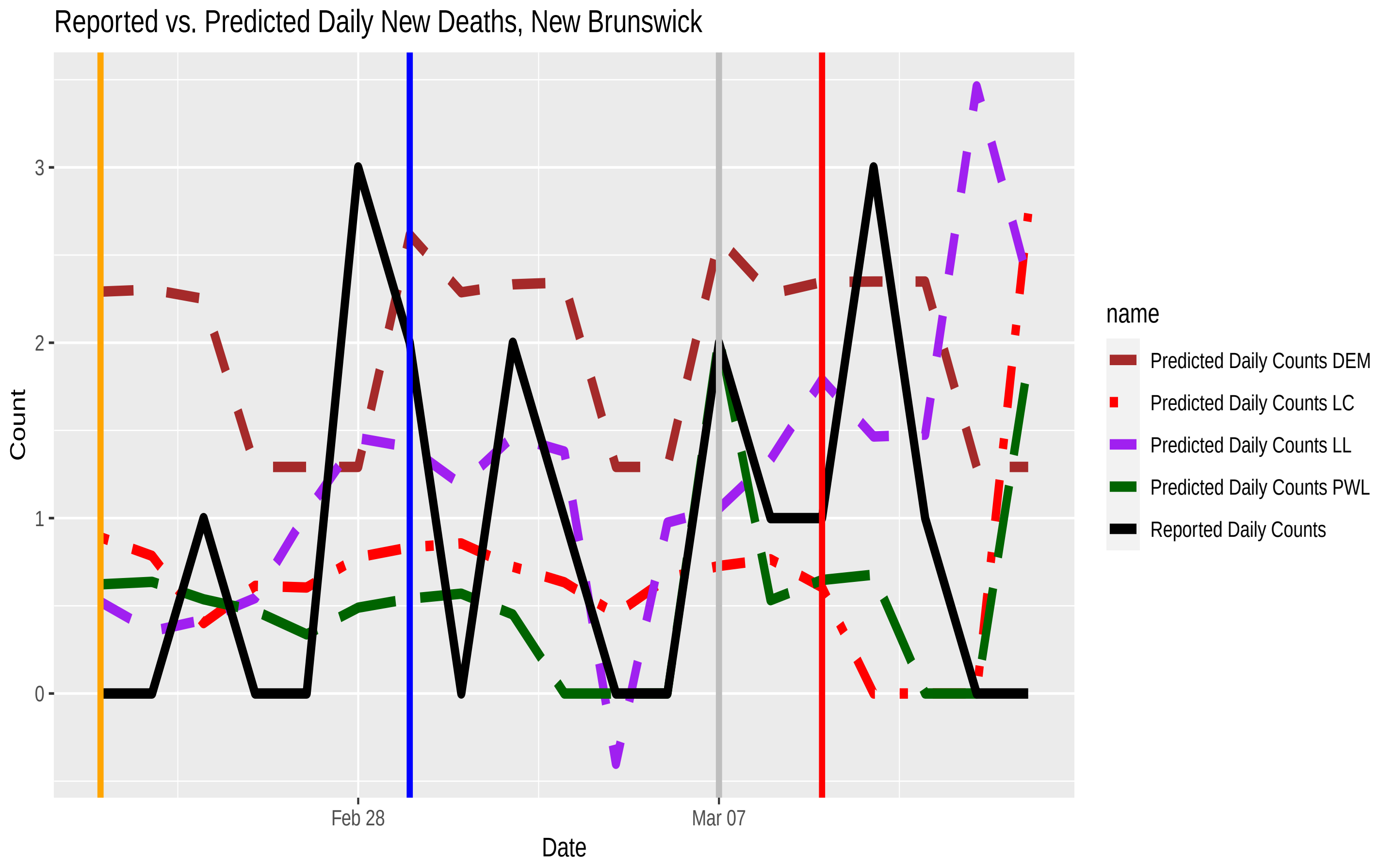

Figure S.3. Prediction for New Brunswick daily reported death counts based on the input data from Nov 28, 2021 to Mar 13, 2022. Vertical lines indicate the start of predictions for each type of model. Mean Squared Prediction Errors (MSPE) for the out-of-sample predictions are as follows. Local Constant: 2 (19 days), Local Linear: 4 (5 days), Piecewise Linear: 1 (13 days), DEM: 0 (7 days).

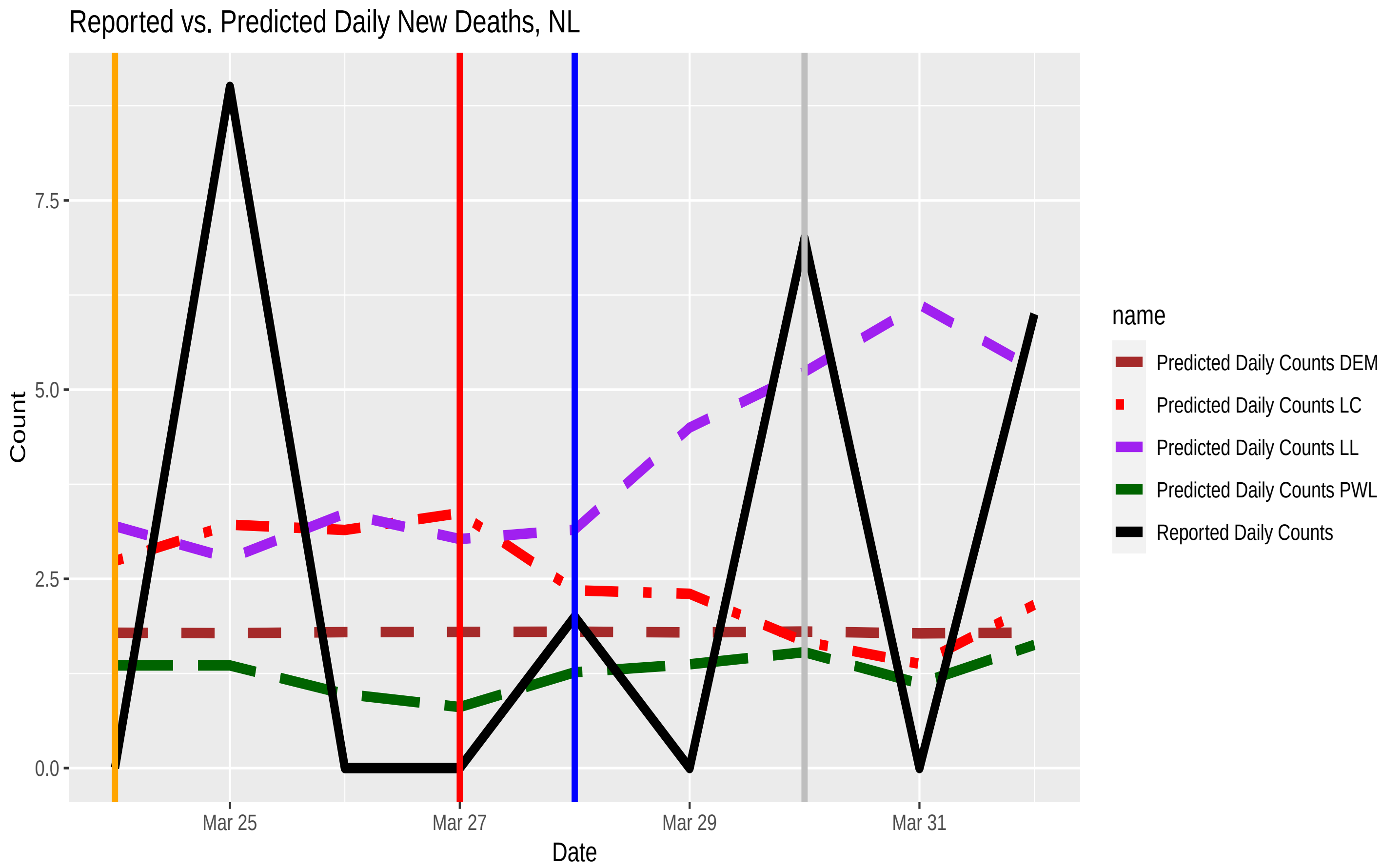

Figure S.4. Prediction for Newfoundland and Labrador daily reported death counts based on the input data from Dec 5, 2021 to April 1, 2023. Vertical lines indicate the start of predictions for each type of model. Mean Squared Prediction Errors (MSPE) for the out-of-sample predictions are as follows. Local Constant: 13 (9 days), Local Linear: 12 (6 days), Piecewise Linear : 11 (5 days), DEM: 22 (3 days).

2 Country Results

Differing results are observed depending on the country data used. For United States and Brazil, local constant methods outperform the local linear methods. Conversely, local linear methods outperform the local constant methods in Israel.

It should be noted that reported death counts for these four countries exhibit periodicity, with a small spike occurring regularly on Mondays (likely due to some deaths from Saturday and Sunday being reported once the work week begins). The mixed results from these four countries illustrate that both local linear and local constant methods should be investigated for each data set in practice.

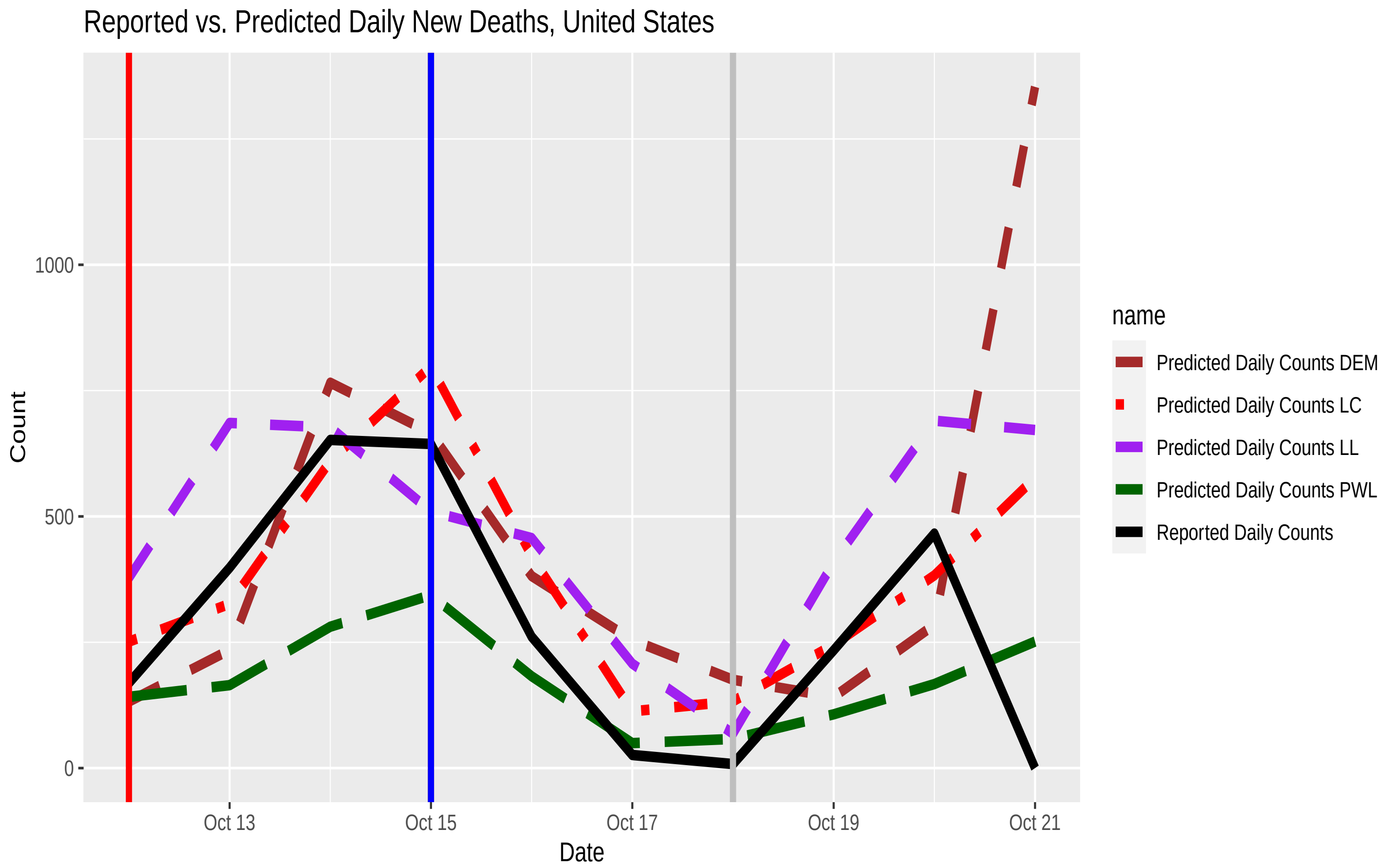

Figure S.5. Prediction results for United States daily reported death counts based on data from Dec 11, 2021 to Oct 21, 2022. Vertical lines indicate the start of predictions for each type of model. Mean Squared Prediction Errors (MSPE) for the out-of-sample predictions are as follows. Local Constant: 58,890 (7 days), Local Linear: 75,209 (10 days), Piecewise Linear: 38,302 (7 days), DEM: 474,746 (4 days).

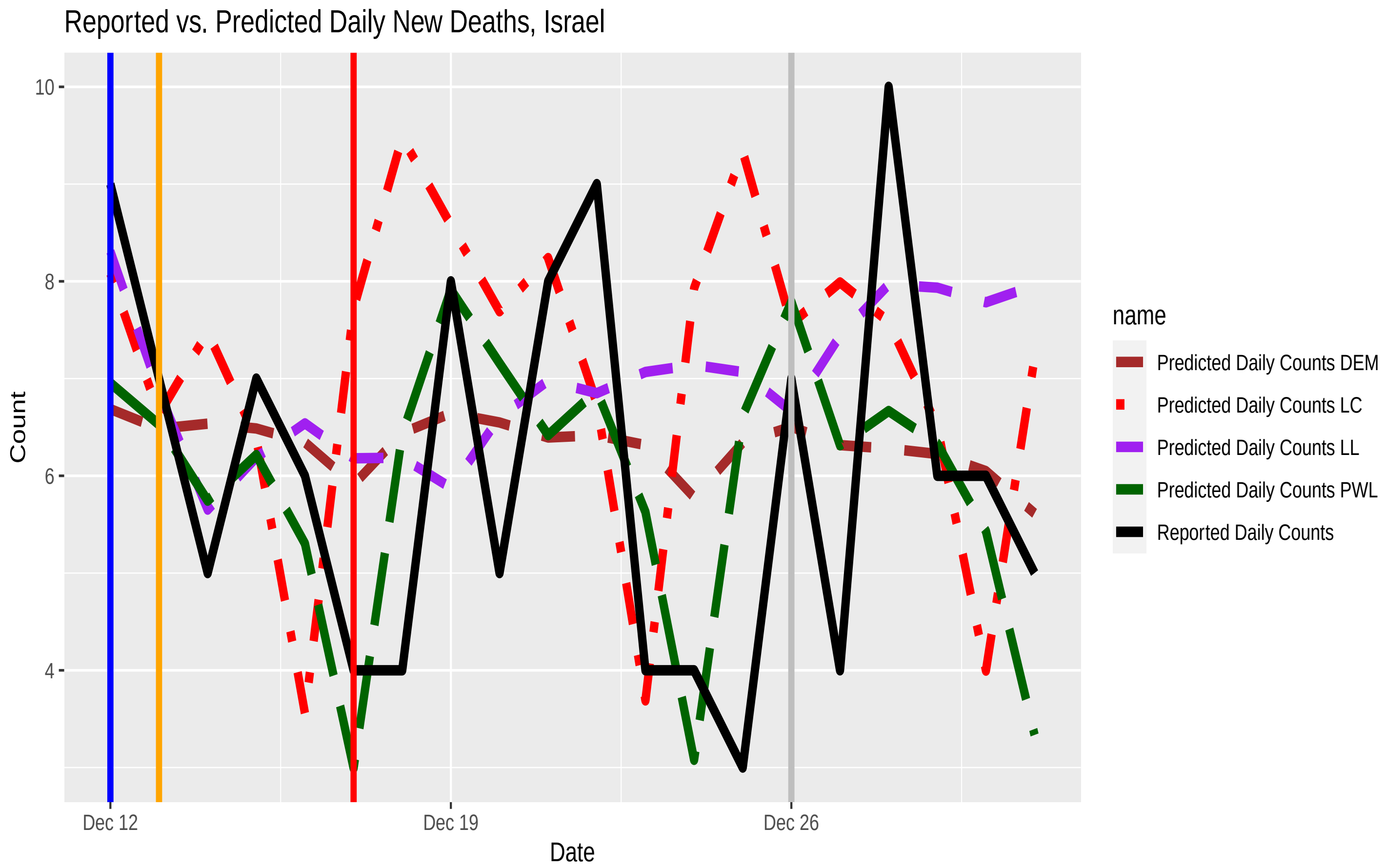

Figure S.6. Prediction for Israel daily reported death counts based on the input data from Dec 30, 2021 to Dec 31, 2022. Vertical lines indicate the start of predictions for each type of model. Mean Squared Prediction Errors (MSPE) for the out-of-sample predictions are as follows. Local Constant: 8 (19 days), Local Linear: 6 (15 days), Piecewise Linear: 3 (20 days), DEM: 5 (6 days).

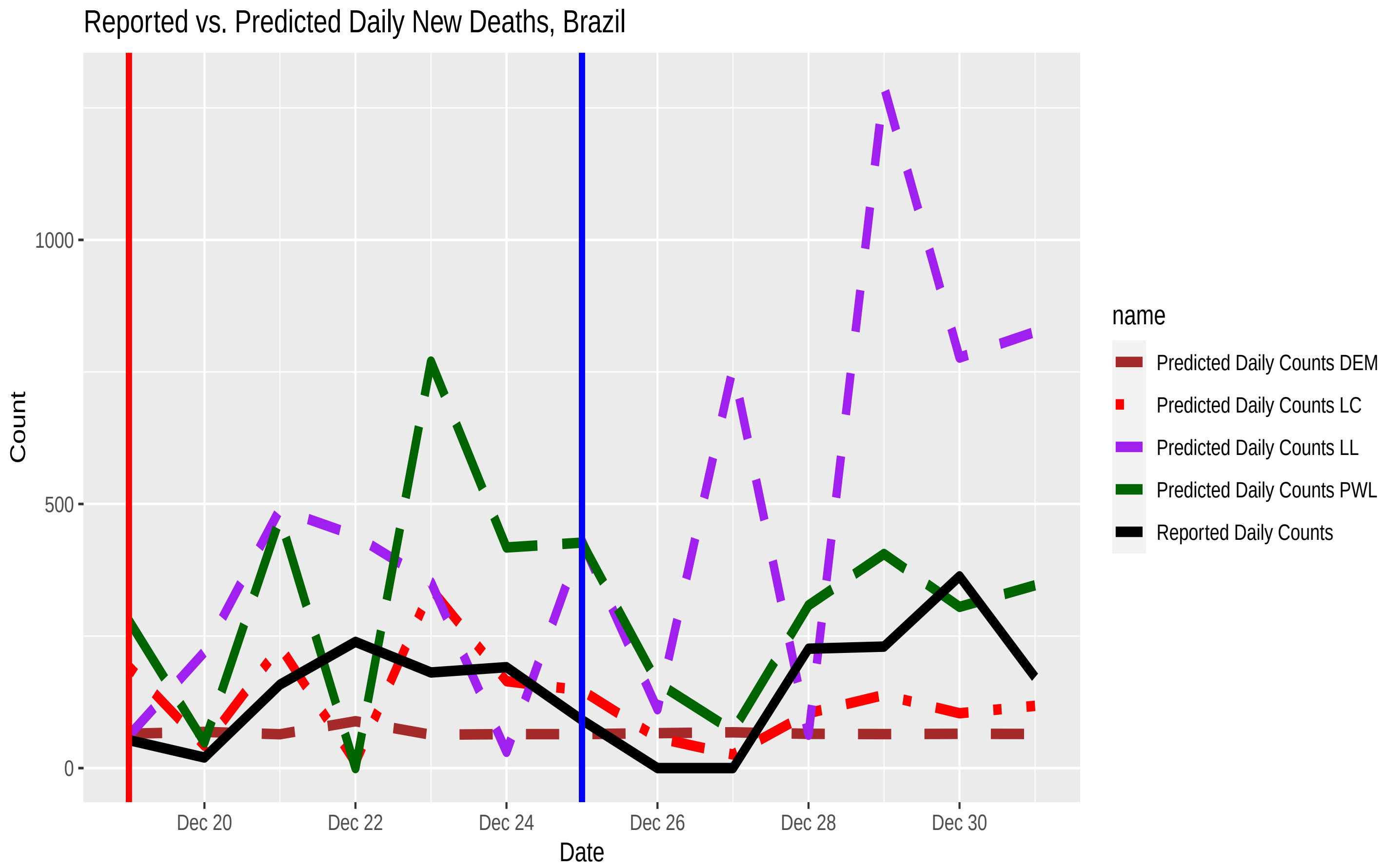

Figure S.7. Prediction for Brazil daily reported death counts based on the input data from Jan 1, 2022 to Dec 31, 2022. Vertical lines indicate the start of predictions for each type of model. Mean Squared Prediction Errors (MSPE) for the out-of-sample predictions are as follows. Local Constant: 14,352 (7 days), Local Linear: 207,468 (13 days), Piecewise Linear: 30,708 (7 days), DEM: 23,536 (7 days).
